# ﻿Diversity of spiders in Daba Mountain National Nature Reserve, Chongqing, China (II): Two new species of Hahniidae (Arachnida, Araneae)

**DOI:** 10.3897/zookeys.1254.154722

**Published:** 2025-10-02

**Authors:** Yu-Jun Cai, Lu-Yu Wang, Ben-Chao Zhu, Wei Lu, Zhi-Sheng Zhang

**Affiliations:** 1 Key Laboratory of Eco-environments in Three Gorges Reservoir Region (Ministry of Education), School of Life Sciences, Southwest University, Chongqing 400715, China Southwest University Chongqing China; 2 Chongqing Urban Ecosystem Positioning Observation and Research Station, Chongqing 400036, China Chongqing Urban Ecosystem Positioning Observation and Research Station Chongqing China; 3 Management Center of Daba Mountain National Nature Reserve, Chongqing 405909, China Management Center of Daba Mountain National Nature Reserve Chongqing China

**Keywords:** Comb-tailed spiders, description, *
Hahnia
*, morphology, *
Sinahahnia
*, taxonomy

## Abstract

Two new species of Hahniidae, *Hahnia dabashan* Cai, Wang & Zhang, **sp. nov.** (♂) and *Sinahahnia chengkou* Cai, Wang & Zhang, **sp. nov.** (♂♀), are described and illustrated with photographs and drawings, based on material from Daba Mountain National Nature Reserve in Chongqing, southwestern China.

## ﻿Introduction

The spider family Hahniidae is relatively small and almost globally distributed, including 242 extant and 16 fossil species, belonging to 29 extant and four fossil genera, with the highest diversity in Europe and Asia (WSC 2025). In China, 48 species in nine genera are known, with most records confined to southwest regions (WSC 2025). Among these, nine species comprising *Hahnia* (4), *Neoantistea* (3) and *Sinahahnia* (2) are known from Chongqing Municipality (Wang et al. 2010; Zhang and Zhang 2013; Liu et al. 2015; Wang and Zhang 2024; Zheng et al. 2025; Wang and Zhang 2025).

This study is part of a series dealing with the diversity of spiders from Daba Mountain National Nature Reserve (MNNR). Following the description of three new species of *Cicurina* Menge, 1871 (Cicurinidae) (Wang et al. 2024), the present paper describes two new species of Hahniidae from the Reserve.

## ﻿Material and methods

Specimens were preserved in 75% ethanol and examined, illustrated, photographed and measured using a Leica M205A stereomicroscope equipped with a drawing tube, a Leica DFC450 camera and Leica Application Suite software (ver. 4.6). Male palps and epigynes were examined and illustrated after dissection. Epigynes were cleared in pancreatin (Álvarez-Padilla and Hormiga 2007). Eye sizes were measured as the maximum dorsal diameter. Prosoma and opisthosoma are measured dorsally. Leg measurements are given as: total length (femur, patella+tibia, metatarsus, tarsus). All measurements are in millimetres. The depository for the material is the Collection of Spiders, School of Life Sciences, Southwest University, Chongqing, China (SWUC). Median apophysis identified here originating from the haematodochal area, follows Burgo et al. (2025), Rubio et al. (2014), Dupérré and Tapia (2024) etc., rather than ‘conductor’ of Gertsch (1934), Bosmans (1992) and Chu et al. (2023).

Abbreviations used in the text: **ALE**, anterior lateral eye; **AME**, anterior median eye; **MOA**, median ocular area; **PLE**, posterior lateral eye; **PME**, posterior median eye.

## ﻿Taxonomy

### ﻿Family Hahniidae Bertkau, 1878

#### ﻿Genus Hahnia

Taxon classificationAnimaliaAraneaeHahniidae

C. L. Koch, 1841

49B7E8A3-8F7A-50FD-88BB-2190288EE325

##### Type species.

*Hahnia pusilla* C.L. Koch, 1841.

##### Composition.

In total, the genus comprises 105 species, among which 26 species are known from China, and four are known from Chongqing: *H. subcorticicola* Liu, Huang & Zhang, 2015, *H. thorntoni* Brignoli, 1982, *H. zhejiangensis* Song & Zheng, 1982 and *H. zhui* Zhang & Chen, 2015 (Wang et al. 2010; Zhang and Zhang 2013; Liu et al. 2015; Zhang and Chen 2015). Here, the fifth *Hahnia* species from Chongqing is described.

#### ﻿ Hahnia dabashan

Taxon classificationAnimaliaAraneaeHahniidae

Cai, Wang & Zhang sp. nov.

7AFD1EAC-6471-545E-B5CA-F04272235EAD

https://zoobank.org/782830A5-9C32-4B69-A987-158300BDD3DF

Figs 1A–C, 2A–D

##### Type material.

***Holotype*** • ♂ (SWUC-T-HA-16-01), China, Chongqing Munic., Chengkou Co., Daba MNNR, Longtian Town, Wuli Vill., 32°04.269'N, 108°39.914'E, elev. 1286 m, 16.03.2018, Z.S. Zhang et al. leg. ***Paratypes*** • 4♂ (SWUC-T-HA-16-02~05), same data as for the holotype.

##### Etymology.

The specific name is derived from the type locality (Dabashan = Daba Mountain); noun in apposition.

##### Diagnosis.

**Figure 1.**
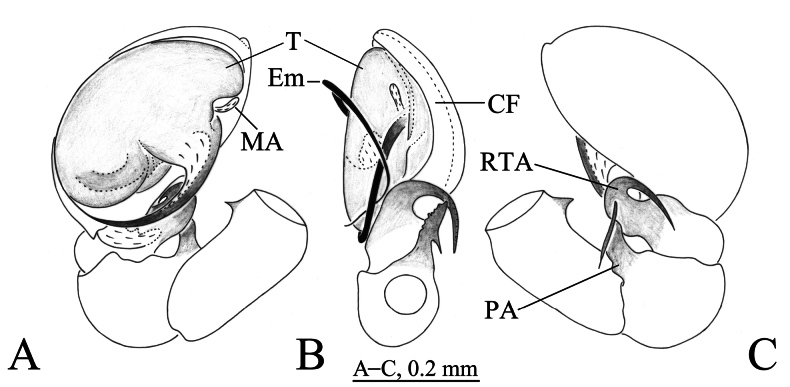
*Hahnia dabashan* sp. nov., male holotype (A, C) and paratype (B). A. Left male palp, ventral view; B. Same, retrolateral view; C. Same, dorsal view. Abbreviations: CF = cymbial furrow; Em = embolus; MA = median apophysis; PA = patellar apophysis; RTA = retrolateral tibial apophysis; T = tegulum.

**Figure 2.**
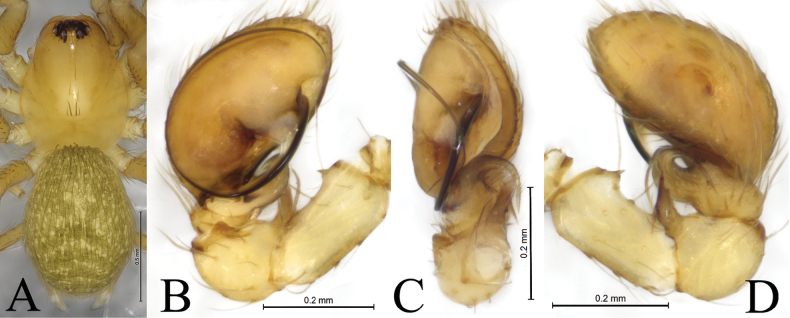
*Hahnia dabashan* sp. nov., male holotype (A, B, D) and paratype (C). A. Male habitus, dorsal view; B. Left male palp, ventral view; C. Same, retrolateral view; D. Same, dorsal view.

The male palp of the new species is similar to that of *H. corticicola* Bösenberg & Strand, 1906 (Liu et al. 2015: 297, fig. 3A–I) in the general shape of the cymbium, embolus, tegulum, and retrolateral tibial apophysis (RTA). It can be distinguished by the following combination of characters: a thorn-like process (TF) on the proximal femur (vs. absent); a flake-like patellar apophysis with a serrated margin (vs. rod-shaped with a hooked tip); and indistinct teeth on the distal retrolateral tibial apophysis (vs. distinct teeth) (Figs 1A–C, 2B–D).

**Figure 3.**
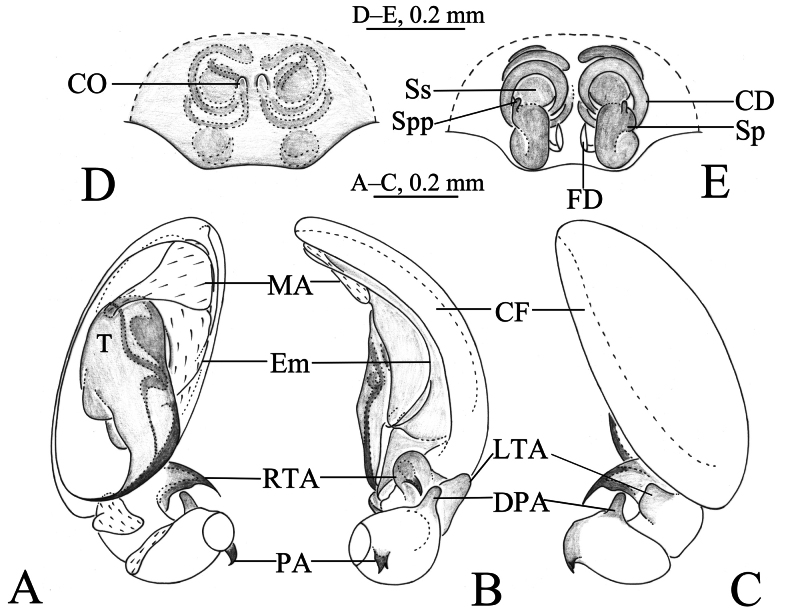
*Sinahahnia chengkou* sp. nov., male holotype (A–C) and female paratype (D–E). A. Left male palp, ventral view; B. Same, retrolateral view; C. Same, dorsal view; D. Epigyne, ventral view; E. Same, dorsal view. Abbreviations: CD = copulatory duct; CF = cymbial furrow; CO = copulatory opening; Em = embolus; FD = fertilization duct; LTA = lateral tibial apophysis; MA = median apophysis; PA = patellar apophysis; RPA = retrolateral patellar apophysis; RTA = retrolateral tibial apophysis; Sp = spermatheca; Spp = spermathecal protuberance; Ss = secondary spermatheca; T = tegulum.

##### Description.

Male holotype (Fig. 2A) total length 1.55. Prosoma 0.78 long, 0.62 wide; opisthosoma 0.84 long, 0.61 wide. Carapace yellowish brown, with few black setae. Eye sizes and interdistances: AME 0.02, ALE 0.05, PME 0.05, PLE 0.0.05; AME–AME 0.02, AME–ALE 0.02, PME–PME 0.05, PME–PLE 0.02, ALE–PLE 0.01. MOA 0.13 long, front width 0.06, back width 0.13. Clypeus height 0.08. Chelicerae yellowish brown, with 2 promarginal and 4 retromarginal teeth. Endites yellowish brown. Labium yellowish brown, wider than long. Leg measurements: I 1.63 (0.50, 0.56, 0.31, 0.26); II 1.55 (0.47, 0.50, 0.31, 0.27); III 1.35 (0.37, 0.45, 0.27, 0.26); IV 1.83 (0.50, 0.64, 0.38, 0.31). Opisthosoma oval, dorsum yellowish brown, dorsally with 3 light chevrons, venter yellowish brown.

***Palp*** (Figs 1A–C, 2B–D). Femur proximal end with thorn-like outgrowth; patellar apophysis (PA) flake-like, 3 times longer than wide in retrolateral view; distal part of retrolateral tibial apophysis (RTA) extending dorsally then proximally; cymbium c. 1.7 times longer than wide, with deep, long (as long as cymbium) and semi-transparent retrolateral furrow; embolus originating mid-retrolaterally (c. 3 o’clock position), clockwise curved along cymbial margin, then inside cymbial furrow in the left male palp; tip of embolus embedded in a transparent pocket. Median apophysis (MA) located near base of embolus.

Female unknown.

##### Variation.

Males (*N* = 5) total length 1.55–1.75.

##### Distribution.

Known only from the type locality in Chongqing, China.

#### ﻿Genus Sinahahnia

Taxon classificationAnimaliaAraneaeHahniidae

Wang & Zhang, 2024

36AB391B-AC35-5711-AD84-E3FBA45FF814

##### Type species.

*Sinahahnia eyu* Wang & Zhang, 2024.

##### Composition.

Three species, all described from China, with two from Chongqing: *S. eyu* Wang & Zhang, 2024 and *S. yintiaoling* Wang & Zhang, 2024 (Wang and Zhang 2024). Here, the third *Sinahahnia* species from Chongqing is described.

#### ﻿ Sinahahnia chengkou

Taxon classificationAnimaliaAraneaeHahniidae

Cai, Wang & Zhang sp. nov.

3847DF55-CC21-5367-BC33-99087F055978

https://zoobank.org/6EC7A5EA-3B6F-46F9-B1C3-CA169719439A

Figs 3A–E, 4A–G

##### Type material.

***Holotype*** • ♂ (SWUC-T-HA-17-01), China, Chongqing Munic., Chengkou Co., Daba MNNR, Longtian Town, Wuli Vill., 32°04.269'N, 108°39.914'E, elev. 1286 m, 16.03.2018, Z.S. Zhang et al. leg. ***Paratypes*** • 2♂9♀ (SWUC-T-HA-17-02~12), same data as holotype • 1♂ (SWUC-T-HA-17-13), Wuli Vill., 32°03.726'N, 108°40.351'E, elev. 1206 m, 16.03.2018, Z.S. Zhang et al. leg. • 1♂2♀ (SWUC-T-HA-17-14~16), Wuli Vill., 32°04.443'N, 108°39.278'E, elev. 1264 m, 16.09.2012, L.Y. Wang, X.K. Jiang leg. • 2♀ (SWUC-T-HA-17-17~18), Xiuqi Town, Xumu Vill., 31°54.484'N, 109°03.556'E, elev. 1670 m, 27.03.2013, X.K. Jiang and X.W. Meng leg.

##### Etymology.

The specific name is derived from the type locality; noun in apposition.

##### Diagnosis.

**Figure 4.**
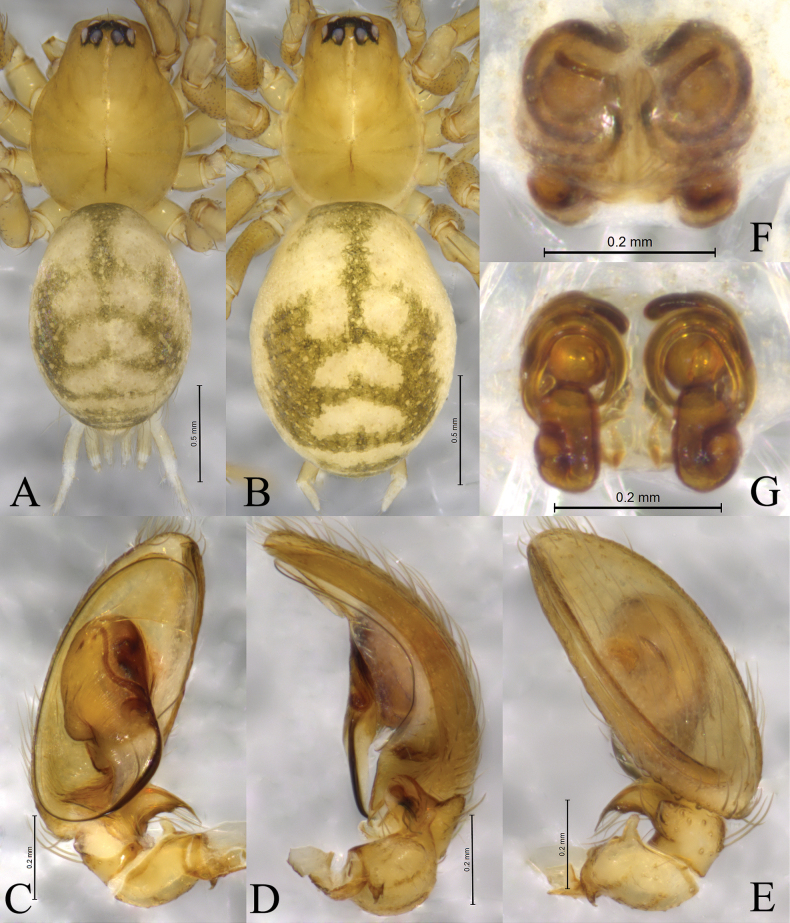
*Sinahahnia chengkou* sp. nov. male holotype (A, C–E) and female paratype (B, F, G). A. Male habitus, dorsal view; B. Female habitus, dorsal view; C. Left male palp, ventral view; D. Same, retrolateral view; E. Same, dorsal view; F. Epigyne, ventral view; G. Same, dorsal view.

The new species resembles *S. eyu* (Wang and Zhang 2024: 251, figs 2, 3C–G, 4B, C) in having a bifurcated patellar apophysis, long, slender embolus, large, membranous median apophysis, and long, spiral copulatory ducts, but differs from the latter by the curved retrolateral tibial apophysis (*vs* twisted with small thorns), the finger-shaped retrolateral patellar apophysis (*vs* absent; Figs 3A–C, 4C–E), and cordate spermathecae (*vs* reniform; Figs 3E, 4G).

##### Description.

Male holotype (Fig. 4A) total length 2.15. Prosoma 1.01 long, 0.80 wide; opisthosoma 1.18 long, 0.84 wide. Carapace yellowish brown. Fovea longitudinal. Eye sizes and interdistances: AME 0.02, ALE 0.07, PME 0.07, PLE 0.09; AME–AME 0.02, AME–ALE 0.01, PME–PME 0.04, PME–PLE 0.02, ALE–PLE 0.01. MOA 0.16 long, front width 0.06, back width 0.19. Clypeus height 0.12. Chelicerae yellowish brown, with 2 promarginal and 4 retromarginal teeth. Endites yellowish brown. Labium yellowish brown. Sternum yellowish brown. Leg measurements: I 2.85 (0.81, 0.91, 0.60, 0.53); II 2.61 (0.77, 0.83, 0.53, 0.48); III 2.08 (0.70, 0.49, 0.44, 0.45); IV 3.07 (0.89, 0.93, 0.72, 0.53). Opisthosoma oval, dorsum yellowish brown, dorsally with 6 light chevrons, venter yellowish brown.

***Palp*** (Figs 3A–C, 4C–E). Femur unmodified. Patella with 2 apophyses − proximal and distal: proximal one (PA) curved, short, with 2 tips, one of them twice larger than other; distal apophysis (DPA) finger-shaped. Tibia slightly shorter than patella, with 2 apophyses: retrolateral apophysis (RTA) curved retrolaterally, with intumescent base and spine-like tip; lateral apophysis (LTA) with blunt end, slightly wider than tall in dorsal view. Cymbial furrow (CF) almost as long as cymbium. Tegulum longer than wide. Embolus with wide embolic base, originating at 6-o’clock position, silk-like, curved clockwise (in left palp) as whole circle, its tip staying inside of cymbial furrow near embolic base. Distal part of median apophysis (MA) almost 7 times wider than its base; sperm duct distinct in tegulum and embolic base, curved inside of embolic base.

Female paratypes total length 2.00–2.47. Paratype (SWUC-T-HA-09-02, Fig. 4B) total length 2.13. Prosoma 0.87 long, 0.63 wide; opisthosoma 1.26 long, 0.95 wide. Carapace yellowish brown. Eye sizes and interdistances: AME 0.03, ALE 0.07, PME 0.07, PLE 0.08; AME–AME 0.02, AME–ALE 0.01, PME–PME 0.05, PME–PLE 0.02, ALE–PLE 0.01. MOA 0.14 long, front width 0.07, back width 0.18. Clypeus height 0.08. Leg measurements: I 11.38 (3.09, 4.21, 2.50, 1.58); II 10.85 (3.04, 3.83, 2.43, 1.55); III 11.19 (2.95, 3.62, 3.09, 1.53); IV 16.17 (3.90, 5.11, 5.10, 2.06).

***Epigyne*** (Figs 3D–E, 4F–G). Plate as long as wide; copulatory opening (CO) small located slightly mid-anteriorly; copulatory duct (CD) thin, long, wrapped 3 times around secondary spermathecae (Ss); secondary spermatheca small, located anteriorly; spermatheca (Sp) about 1.5 times longer than wide, folded in half, as large as secondary spermatheca, with a small protuberance antero-laterally (Spp); spermathecae spaced slightly wider by one width, but narrower than the length of it.

##### Variation.

Males (*N* = 5) total length 2.08–2.22, females (*N* = 13) total length 2.00–2.47.

##### Distribution.

Known only from the type locality in Chongqing, China.

## Supplementary Material

XML Treatment for Hahnia

XML Treatment for Hahnia dabashan

XML Treatment for Sinahahnia

XML Treatment for Sinahahnia chengkou

## ﻿Additional information

### Use of AI

No use of AI was reported.

### Author ORCIDs

Yu-Jun Cai https://orcid.org/0009-0003-8259-6783

Lu-Yu Wang https://orcid.org/0000-0002-5250-3473

Ben-Chao Zhu https://orcid.org/0009-0007-1235-8308

Wei Lu https://orcid.org/0009-0005-3504-3777

Zhi-Sheng Zhang https://orcid.org/0000-0002-9304-1789

### Data availability

All of the data that support the findings of this study are available in the main text.
